# Investigation of microbial coinfection in 453 septic COVID-19 patients admitted to hospital; a retrospective study

**DOI:** 10.2144/fsoa-2023-0066

**Published:** 2023-07-29

**Authors:** Armin Khavandegar, Zeinab Siami, Sogand Goudarzi, Aziz Rasooli, Yeganeh Ettehad

**Affiliations:** 1 Sina Trauma & Surgery Research Center, Sina Hospital, Tehran University of Medical Sciences, Tehran, Iran; 2 Infectious Disease Department, Alborz University of Medical Sciences, Karaj, Iran; 3 Department of Infectious Diseases & Tropical Medicine, Ziaeian Hospital, Tehran University of Medical Sciences, Tehran, Iran; 4 Department of Anesthesiology & Perioperative Medicine, Tufts Medical Center, Tufts University School of Medicine, Boston, MA, USA; 5 Department of Emergency Medicine, Faculty of Medicine, Tehran University of Medical Sciences, Tehran, Iran; 6 Student Research Committee, Alborz University of Medical Sciences, Karaj, Iran

**Keywords:** bacterial coinfection, coinfection, COVID-19, SARS-CoV-2, underlying disease

## Abstract

**Aim:**

We evaluated the rate of COVID-19 microbial coinfection in an Iranian population.

**Methods:**

In this single-center, retrospective observational study, we evaluated 453 septic COVID-19 patients for possible coinfection in an Iranian hospital.

**Results:**

Overall, 211 (46.57%) cases died due to COVID-19 complications. Positive respiratory secretion and blood cultures were reported in 99 (21.9%) and 19 (4.2%) cases. *Klebsiella* species were the most commonly isolated microorganisms in respiratory (n = 50, 50.5%) and blood (n = 10, 52.6%) specimens. After adjustment for underlying disorders, positive respiratory microbial cultures significantly increase the odds of developing death, intubation, and ICU admission and negatively impact healthy discharge (P < 0.05).

**Conclusion:**

Coinfections with bacteria and fungi independently contribute to poor outcomes in septic COVID-19 patients.

## Plain language summary

COVID-19 bacterial/fungal coinfection is associated with severe mortality rates as it complicates the primary viral infection. This study evaluated 453 patients admitted to an Iranian hospital with COVID-19 and concomitant sepsis for microbial coinfection. A total of 99 (21.9%) cases had positive respiratory secretion cultures, and 19 (4.2%) had positive blood cultures. *Klebsiella* species were the most commonly yielded microorganism in both respiratory (n = 50, 50.5%) and blood (n = 10, 52.6%) specimens. Bacterial and fungal microbial coinfection are independent determinants of poor outcomes in septic COVID-19 cases.

SARS-CoV-2 is the main infectious agent responsible for COVID-19 [1]. Over 767 million infected cases, including over 6.9 million deaths, have been reported [2].

A disturbing concern in the COVID-19 era and its management is coinfection, with bacteria being the main coinfection organism [3]. Bacterial coinfection is problematic for COVID-19 patients as it complicates the primary viral infection, worsens the prognosis, and remarkably increases the mortality rate [4,5].

COVID-19 bacterial coinfection rate varies widely geographically, as it is reported at 3.6% in New York in patients admitted between March and April 2020, with a median age of 62 [6], 7.2% in Barcelona in patients admitted between February and April 2020, with a median age of 62 years [7], and 28% in a hospital in France in patients admitted between March and April 2020, with a median age of 61 years [8]. Overall, the prevalence of coinfection in COVID-19 and its effect on mortality have been poorly investigated [9]. Results from China demonstrated that bacterial coinfection in adult COVID-19 cases ranged from 0–16.7% [10]. A large cohort of over 12,000 samples in the US revealed a bacterial coinfection rate of 55.4% in COVID-19-positive cases [11]. Specifically, a pooled estimated prevalence of bacterial coinfection in respiratory cultures was 5.20% [12].

Besides, fungal coinfection has been reported in many previous studies [6,13–17]. A fungal coinfection rate of 32.8%, with most of them being Candida species, has been reported in the literature [15]. As a result of immune dysregulation, alteration of intestinal microbiota, and underlying comorbidities, admitted SARS-CoV-2 individuals are remarkably prone to fungal infections [18,19]. It is well-established that unstable COVID-19 cases have a higher rate of microbial and, more specifically, fungal coinfection [13]. The widespread use of steroidal medicines and antibodies, coupled with the virus' immune dysregulation, can exacerbate existing fungal infections and lead to a greater risk of secondary infections in patients with COVID-19 [20].

Although many studies have evaluated bacterial coinfection in COVID-19 cases, few were devoted to septic cases. In prior studies, reporting the incidence of coinfections, various cultures would not have been drawn, and sepsis was not on the differential diagnosis for these patients. The aim of this study was to investigate the association between concomitant sepsis in adults with COVID-19 and clinical outcomes and the potential risk factors for developing sepsis during COVID-19.

## Materials & methods

### Subjects & variables

This single-center, retrospective study evaluated 7134 patients who were admitted to the Imam Ali Hospital, Karaj, Iran, between March 2020 and October 2021, with an initial concomitant presentation of COVID-19 and sepsis. Diagnosis of COVID-19 was determined by the presence of fever (temperature >38°C) and/or Respiratory Rate (RR) >20 in the setting of SARS-CoV-2 positivity on RT-PCR from a nasopharyngeal swab [21]. As the presence of fever and/or tachypnea are also diagnostic criteria for sepsis, we defined sepsis by the presence of fever and/or tachypnea, at least one of the other systemic inflammatory response syndrome criteria (heart rate >90 beats per minute; white blood cell count (WBC) >12 or <4 × 10^3^/μl or >10% band cells [21]), and a suspected or confirmed infection other than SARS-CoV-2. We excluded patients younger than 18 years old, outpatient cases, and those who developed sepsis after the time of hospital admission. Five main outcomes were evaluated in this study: ICU admission, mechanical ventilation, in-hospital death, the status of health at discharge, and hospital readmission. This study is approved by the Research Ethics Committee of Alborz University of Medical Sciences with approval ID: I.R.ABZUMS.REC.1400.080.

### Sample size

Considering “death” as the main outcome in COVID-19 cases, after reviewing the literature, a series of sessions with the expert panel, and power calculation, approximately 390 patients were suggested to be sufficient to detect the suspected difference in effect. However, considering all confounding factors, to make sure to draw a justified and reliable conclusion, we have included 453 cases in this study.

### Medical history & laboratory measurement

Patient comorbidities were recorded and incorporated into multivariate analyses (see Statistical Analysis section below). Within 48 hours of admission, sputum, endotracheal aspirates, nasopharyngeal and oropharyngeal swabs, and blood samples were collected, utilizing containers based on the standard protocol developed by the CDC's recommendations for collection, transportation, and processing of specimens [22]. Samples of respiratory and blood cultures were then sent to the laboratory.

### Statistical analysis

Data in normal distribution are represented as mean ± standard deviation; otherwise, they are described as median ± interquartile range. Nominal and categorical data are presented as frequency (%). The association between variables with five main outcomes in this study, i.e., death, ICU admission, mechanical ventilation, readmission, and healthy discharge, was assessed using binary logistic regression models. All statistical analyses were conducted by SPSS version 22. A p-value of less than 0.05 is considered statistically significant in all analyses.

## Results

### Demographic characteristics & clinical outcomes

Of the 453 patients, 254 (56.1%) were male, and 199 (43.9%) were female. The average age was 63.38 and 63.17 years for males and females, respectively. Thirty (6.6%) patients were hospitalized for 1–3 days, 129 (28.5%) patients for 4–7 days, 155 (34.2%) patients for 8–17 days, and 139 (30.7%) patients for at least 18 days. ICU admission was documented for 209 (46.13%) cases. The average age of ICU-admitted cases was 63.82 ± 16.93. Two hundred forty-one (53.20%) patients were discharged from the hospital with a mean age of 61.6 ± 17.35; 18 cases were readmitted. 211 (46.57%) cases died due to COVID-19 complications, with an average age of 65.42 ± 17.45. Finally, 209 (46.13%) patients were intubated at least once during hospitalization at the age of 64.64 ± 17.04. Patients' demographic characteristics and clinical outcomes are presented in Table 1.

**Table 1.** Characteristics of demographic, laboratory, and clinical findings of septic COVID-19 cases (n = 453).

Age; mean ± SD; years	63.29 ± 17.46
Male; n (%)	199 (43.9%)
Underlying disease; n (%)	
Hypertension	194 (42.82%)
Ischemic heart disease	126 (27.81%)
Diabetic mellitus	119 (26.26%)
Renal disorders	42 (9.27%)
Opioid addiction	22 (4.85%)
Hepatic disorders	18 (3.97%)
Cancer	15 (3.31%)
Cultures; n (%)	
Respiratory secretions culture	99 (21.9%)
Blood culture	19 (4.2%)
Laboratory findings; n (%)	
Leukopenia (<4 × 10^3^ cell/mm^3^) or Leukocytosis (>11 × 10^3^ cell/mm^3^)	333 (73.5%)
Elevated CRP (qualitative; >10 mg/l)	397 (87.6%)
Elevated ESR (qualitative; >15 mm/h in men and >20 mm/h in women)	388 (85.7%)
Elevated Procalcitonin (>0.50 ng/ml)	83 (18.3%)
Elevated D-dimer (>0.5 mcg/ml)	446 (98.5%)
Hospital Length of Stay (LOS); n (%)	
1–3 days	30 (6.6%)
4–7 days	129 (28.5%)
8–17 days	155 (34.2%)
≥18 days	139 (30.7%)
Outcomes; n (%)	
Death	211 (46.57%)
ICU admission	209 (46.13%)
Intubation	209 (46.13%)
Readmission	18 (3.9%)
Healthy discharge	244 (53.9%)

ESR: Erythrocyte sedimentation rate; ICU: Intensive care unit; LOS: Length of stay; SD: Standard Deviation.

### Underlying diseases

Hypertension was the most common comorbidity, reported in 194 (42.82%) cases, while opioid addiction was the less common underlying disorder, observed in 22 (4.85%) patients. Ischemic hearts diseases, diabetes mellitus, renal disorders, opioid addiction, hepatic disorders, and cancer lay in between, reported in 126 (27.81%), 119 (26.26%), 42 (9.27%), 22 (4.85%), 18 (3.97%), and 15 (3.31%) cases, respectively.

ICU admission was significantly higher in patients with “hypertension (p: 0.001)”, and “ischemic heart diseases (p: 0.007)”. Death was significantly higher in cases with “diabetes mellitus (p: 0.004)”, “hypertension (p: 0.002)”, “cancer (p: 0.002)”, “renal disorders (p: 0.048)”, and “opioid addiction (p: 0.050)”. Readmission had no significant association with each of the comorbidities (p > 0.05). Healthy discharge was significantly associated with diabetes mellitus (p: 0.004), hypertension (p: 0.002), cancer (p: 0.007), renal disorders (p: 0.033), and opioid addiction (p: 0.047). Intubation was significantly higher in patients with diabetes mellitus (p: 0.001) and hypertension (p: 0.000). The association of underlying conditions and patients' outcomes are summarized in Table 2.

**Table 2.** Univariable logistic regression of the association between underlying diseases and cultures and five main patients' outcomes.

	ICU admission		Death		Readmission		Healthy Discharge		Intubation	
	OR (95% CI)	p-value	OR (95% CI)	p-value	OR (95% CI)	p-value	OR (95% CI)	p-value	OR (95% CI)	p-value
Diabetic mellitus	1.13 (0.74–1.72)	0.545	1.83 (1.20–2.80)	0.004^‡^	0.7 (0.23–2.24)	0.584	0.53 (0.35–0.81)	0.004^‡^	2.094 (1.36–3.20)	0.001^‡^
Hypertension	1.83 (1.26–2.67)	0.001^‡^	1.78 (1.22–2.59)	0.002^‡^	0.76 (0.29–1.97)	0.578	0.55 (0.38–0.81)	0.002^‡^	2.25 (1.53–3.28)	0.000^‡^
Ischemic heart disease	1.76 (1.6–2.67)	0.007^‡^	1.33 (0.88–2.01)	0.163	1.89 (0.74–4.83)	0.174	0.73 (0.48–1.10)	0.138	1.45 (0.96–2.19)	0.074
Cancer	2.33 (0.78–6.94)	0.117	7.66 (1.70–34.36)	0.002^‡^	1.04 (1.02–1.06)	0.411	0.20 (0.05–0.73)	0.007^‡^	1.32 (0.47–3.71)	0.595
Renal disorders	0.69 (0.36–1.30)	0.254	1.88 (0.99–3.56)	0.048^†^	1.09 (0.24–4.91)	0.903	0.50 (0.26–0.95)	0.033^†^	1.16 (0.62–2.16)	0.633
Hepatic disorders	0.42 (0.15–1.21)	0.099	1.41 (0.55–3.65)	0.472	1.36 (0.17–10.81)	0.769	0.67 (0.26–1.74)	0.414	1.15 (0.45–2.96)	0.767
Opioid addiction	0.42 (0.14–1.20)	0.456	2.50 (0.99–6.25)	0.050	1.36 (0.17–10.81)	0.315	0.38 (0.15–0.95)	0.047^†^	1.15 (0.44–2.96)	0.101
Respiratory secretion culture	1.74 (1.11–2.73)	0.015^†^	6.91 (4.01–11.91)	0.000^‡^	0.95 (0.30–2.93)	0.931	0.14 (0.08–0.25)	0.000^‡^	12.11 (6.50–22.58)	0.000^‡^
Blood culture	2.00 (0.77–5.19)	0.145	6.35 (1.82–22.13)	0.001^‡^	1.04 (1.02–1.06)	0.353	0.15 (0.04–0.52)	0.001^‡^	4.55 (1.48–13.94)	0.004^‡^

† Statistically significant.

‡ Highly statistically significant.

ICU: Intensive care unit; OR: Odds ratio.

### Cultures

**Figure 1.**
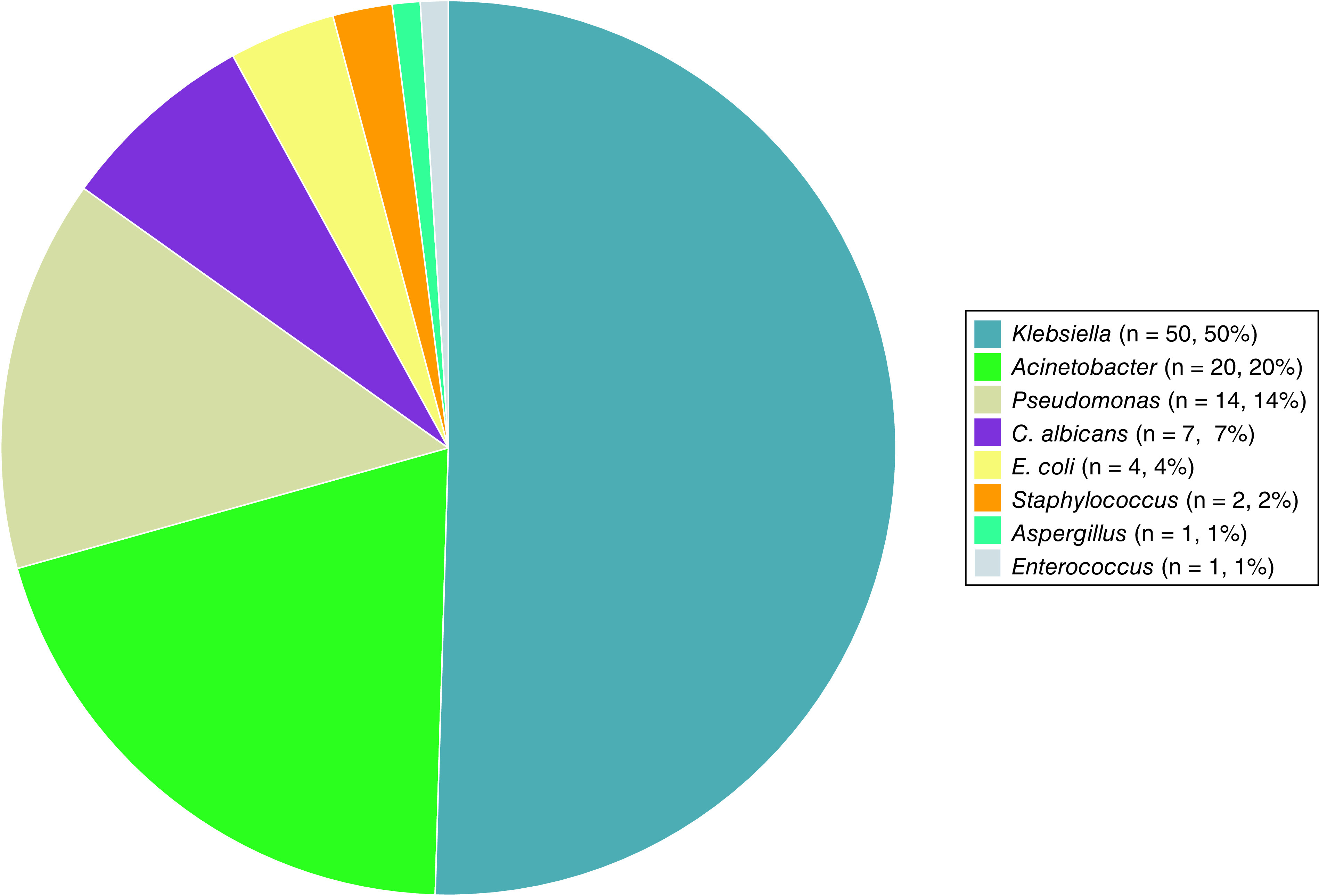
Details of positive cultures based on types of microorganisms in respiratory secretion cultures.

Of the 453 septic COVID-19 patients, 99 (21.9%) cases had positive respiratory secretion cultures, and 19 (4.2%) had positive blood cultures. In respiratory secretions, *Klebsiella* species were the most common form (n = 50, 50.5%), followed by *Acinetobacter* species (n = 20, 20.2%), *Pseudomonas* species (n = 14, 14.1%), and *Candida albicans* (n = 7, 7.1%), *Escherichia coli* (n = 4, 4%), *Staphylococcus* species (n = 2, 2%), *Enterococcus* and *Aspergillus* species (both n = 1, 1%). *Klebsiella* species were also the most common organism in positive blood culture samples (n = 10, 52.6%). We have found no fungal coinfection in blood samples. No concurrent bacterial and fungal coinfection was reported in this study. Details of positive respiratory cultures based on the types of microorganisms are depicted in Figure 1.

Positive respiratory cultures increased the odds of intubation by 12.11-times (p: 0.001), death by 6.94-times (p: 0.001), and ICU admission by 1.74 (p: 0.015). Positive blood culture increased the odds of intubation 4.55-times (p: 0.004), and death 6.35-times (p: 0.001) (Table 2).

After adjustment for all underlying diseases, age, and gender, multiple logistic regression revealed that positive respiratory microbial culture significantly increased the odds of developing poorer outcomes, except for readmission. The results were almost similar for the positive blood cultures. Positive blood specimens significantly increased the odds of developing poorer outcomes, except for readmission and ICU admission. The multiple regression model of the association between septic COVID-19 cases cultures and patients' outcomes after adjustment for underlying disorders is demonstrated in Table 3.

**Table 3.** Multiple regression model of the association between septic COVID-19 cases cultures and patients' outcomes after adjustment for underlying disorders, age, and gender.

	ICU admission		Death		Readmission		Healthy discharge		Intubation	
	OR (95% CI)	p-value	OR (95% CI)	p-value	OR (95% CI)	p-value	OR (95% CI)	p-value	OR (95% CI)	p-value
Respiratory secretion culture	1.68 (1.05 to 2.70)	0.029^†^	9.23 (5.09 to 16.75)	<0.001^†^	1.00 (0.32 to 3.13)	0.998	0.11 (0.07 to 0.20)	<0.001^‡^	14.88 (7.65 to 28.93)	<0.001^†^
Blood Culture	1.76 (0.65 to 4.73)	0.262	6.08 (1.60 to 23.06)	0.008^‡^	1.02 (0.98 to 1.06)	0.998	0.15 (0.04 to 0.56)	0.005^‡^	6.29 (1.80 to 21.98)	0.004^‡^

† Statistically significant.

‡ Highly statistically significant.

ICU: Intensive care unit; OR: Odds ratio.

## Discussion

Antimicrobial therapy in COVID-19-infected cases for either a suspected or a confirmed respiratory coinfection or superinfection is beneficial in patient management. In our study, the frequency of antibiotic regimens in non-septic COVID-19 hospitalized patients was 76%, while it was 99% in septic COVID-19 patients. The mortality rate in all hospitalized COVID-19 patients was nearly 15%, and in septic COVID-19 patients were 46%. Besides, the mortality rate in patients with a positive culture was 81%. In other studies, the prevalence of targeted or suspected antimicrobial therapy varied widely, from 58% in Guan's study [23] to 99% in a study in China [24].

In a comprehensive review article of nine original articles with a report on coinfection, of 806 COVID-19 patients, 62 (8%) cases of bacterial/fungal coinfection were reported, while 1450/2010 (72%) received antimicrobial therapy [19]. In a study by Goyal *et al.* on COVID-19 patients in March 2020 with a median age of 62.2 years, 19 of the 338 cases (6%) of COVID-19 yielded microbial coinfection [25]. In another study by Wang *et al.*, 7% of patients (5 of 69) had positive respiratory secretion cultures [26]. In a recent study of 161 pediatric SARS-CoV-2 Omicron BA.2 confirmed cases, 24 (14.9%) cases had bacterial coinfection [27]. Besides, a coinfection rate of 8% (14 out of 185 COVID-19 cases) was reported in the Jordanian population [28]. In a comprehensive meta-analysis to draw out bacterial coinfection prevalence in Eastern Mediterranean Regional Office (EMRO) and South-East Asia Regional Office, evaluating almost 55,000 COVID-19 cases, a total pooled estimated bacterial prevalence of 20.97% was yielded, and almost a quarter of them were respiratory subtypes [12].

In most studies, the authors did not identify the patients' health status; in other words, microbial coinfection frequency has not been reported separately in critical and non-critical COVID-19 cases. In our study, we excluded those patients developing sepsis during hospitalization and only included 453 septic COVID-19 patients on admission. Of them, 118 (26.1%) had positive microbial coinfection on admission, including 21.9% and 4.2% positive sputum and blood cultures, respectively. Clearly, there is a great difference in the amount of microbial coinfection in our study compared with those previously reported. A possible reason is that we only included septic COVID-19 patients, despite other studies. Hence, underlying microbial coinfection could be a reasonable rationale for septic COVID-19 patients on admission. Accordingly, in some cohorts, the coinfection rate, including viral coinfections, was reported to be 0–3% [29–31], while in others, it was up to 20% of patients [32]. Some authors believe this heterogeneous coinfection rate is a spatiotemporal variation in viral characteristics [33]. Another possible reason for higher rates of microbial coinfection in Iranian COVID-19 septic patients could be a higher rate of comorbidities.

This study revealed that *Klebsiella* species was the foremost microbial organism isolated from respiratory and blood samples. In a large multicenter cohort of over 88,000 COVID-19 cases in the UK and the US, *Staphylococcus* was the most common pathogen with a 24–33% prevalence [34]. Besides, Contou *et al.* [8] revealed that Methiciline-sensitive *Staphylococcus aureus* was observed in ten of 31 cases with bacterial coinfection. In a meta-analysis by Musuuza *et al.* [14], *Klebsiella*, *Streptococcus*, and *Staphylococcus* comprised 30% of bacterial coinfection. Altogether, remarkable heterogeneity in the distribution of coinfection microbial agents was observed in the SARS-CoV-2 infection setting [10].

The risk of developing acute coronary syndrome (ACS) in COVID-19 hospitalized patients is reported at 1.7%, while in our study [35], 8 (1.8%) developed ACS. The risk of developing Acute Kidney Injury (AKI) varies differently in studies, from 3% to 15% [5,36]. In this survey, 42 (9.3%) patients developed AKI. The frequency of neurological manifestations, including headache, dizziness, ataxia, altered sense of smell, and seizure, were seen in up to 45.5% of cases of severe infection [37]. In our study, neurological symptoms, excluding headaches, were observed in 33 (7.3%). In Poissy *et al.'s* survey, pulmonary embolism (PE) in ICU-admitted COVID-19 patients was 20.6%, while in this study, only three patients (0.7%) developed PE [38].

Hypertension is believed to be the most frequent medical condition in hospitalized cases in 49.7% of COVID-19 patients, followed by diabetes mellitus (28.3%) and cardiovascular diseases (27.8%) [39]. Malignancy (1.5%), renal disorders (0.8%), and immunodeficiency stated (0.01%) are the next more frequent medical conditions in COVID-19 patients, either hospitalized or outpatients [40]. In our survey, hypertension was the most common comorbidity state (42.8%), followed by ischemic heart disease (27.8%), Diabetes Mellitus (26.2%), renal disorders (9.2%), opioid addiction (4.8%), hepatic disorders (3.9%) and malignancy state (3.3%).

In a study of 52 critically ill patients with a mean age of 59.7 years with a confirmed COVID-19 in China admitted by January 2020, it was reported that 32 (61.5%) of them died within 28 days, 37 (71%) cases required mechanical ventilation [1]. In our study, consisting of septic patients with a mean age of 63.29, 209 (46.1%) cases were admitted to ICU, and 209 (46.1%) cases required mechanical ventilation. A total of 241 (53.2%) were discharged from the hospital, and 211 (46.1%) patients died during hospitalization.

In this study, we thoroughly evaluated 453 septic COVID-19 patients. Sputum and blood cultures were taken in the first 48 hours of admission, and eventually, patients' outcomes were analyzed separately based on underlying disorders and laboratory results. The data in this article originated from a single center, which may limit the generalizability of the effect. Due to the rapidly evolving nature of COVID-19, further studies are needed to investigate the findings in novel species. Besides, as SIRS efficacy in predicting COVID-19 patients' outcomes seemed to fail compared with other predicting scores [21], in this study, we applied an adapted version of SIRS, as described in the methods section, to encompass more severe cases. Further studies are needed to evaluate the effectiveness of our adapted SIRS version in predicting COVID-19 patients' outcomes.

## Conclusion

In this retrospective analysis of a large cohort of Iranian patients with COVID-19 and concomitant sepsis, the incidence of respiratory microbial coinfection was nearly five-times that of bloodstream coinfection. *Klebsiella* species were the most commonly extracted microbial coinfection from both respiratory and blood samples. Fungal coinfection was only found in respiratory samples. Bacterial and fungal microbial coinfection are independent determinants of developing poor outcomes in septic COVID-19 cases.

**Summary points**

- Microbial Coinfection in septic COVID-19 cases has not yet been fully established.
- Microbial coinfection can exacerbate SARS-CoV-2 cases min outcomes.
- The main outcome that can be impacted mostly by microbial coinfection is mechanical ventilation.
- *Klebsiella* species are the most common organism yielded from both respiratory and blood samples in septic SARS-CoV-2 cases.
